# Paying reviewers for scientific papers and ethical committees

**DOI:** 10.1590/S1679-45082014ED3259

**Published:** 2014

**Authors:** Jacyr Pasternak, Sidney Glina

**Affiliations:** 1 Hospital Israelita Albert Einstein, São Paulo, SP, Brazil.

Richard Smith, former editor of the British Medical Journal, says peer review is hard to define in operational terms,^(1)^ but that almost everybody agrees that it is at the heart of the practice of science. There are many ways of doing it. The editor, for example, can send the paper to two friends who are or are not aware of the subject. If both are favorable to publication of the paper, it will be published. If both advise against publication the editor makes a decision or sends the paper to a third friend to settle the issue (when editors begin their career they have many friends, but they end up losing a lot of them after some time in the business). In Smith’s paper on peer review published in the Journal of the Royal Society of Medicine, he mentions Robbie Fox, a former editor of the Lancet, who was no admirer of peer review system and who joked that the Lancet chooses articles to publish by throwing all papers received down the stairs and publishing those that reached the bottom. In addition, Dr Smith stated that “a systematic review of all evidence on peer review concluded that the practice of peer review is based mostly in faith rather than facts…”

We all know the problems with peer review. It is slow: those who agree to do it, do so in their free time. Most of reviewers do not have a lot of free time, as they are overwhelmed with requests for do more peer review among other tasks. It’s inconsistent: some papers submitted to one journal are considered excellent, whereas in other journals are not even considered to be peer reviewed. Many biases exist and they are not prevented by anonymous review. In fact, if reviewers are well chosen and are in the right area, they can easily identify authors or at least the institution where they work. Some authors have a personal style, and if you read the paper you will be able to identify them. If authors are from less prestigious institutions – for instance, being in Latin America rather than in a developed nation – there is the Matthew effect: “to those who have all, all shall be given; to those that have not, the little they have will be taken from them”. In other words, people from those institutions and individuals not known to the reviewers have more difficulties getting published. Negative results are hard to publish, even when they confront conventional knowledge, i.e., things everybody knows are correct, but have never been, and sometimes they are not that actually correct.

Regardless, we are stuck with peer review. Like democracy: it is a terrible system but better than all alternatives tested to it. Many suggestions have been made to improve the peer review system and one that should be considered is paying reviewers for their work. Reviewers should be professionals and trained; nobody is born a reviewer. Paper reviewing takes time and requires a lot of work. The argument against paying reviewers sees science as involving a community of scholars who do a review today in order to get a review another day, both for free. As many things, such as “free medical care”, things seems that to be free are not, and in the end somebody always pays the bill.

Many journals charge scientists when their papers are reviewed, but few journals pay their reviewers. This happens mostly, but not only, with papers submitted to open access journals. If we consider reviewers as professionals, they should be educated to do the job, and yes, they should be paid. Total pay can be modest, and reviewers would not make a living from it, but they would be accountable, and if they get paid, more careful work will be done and deadlines will be respected (reviews that consist of comments such as “excellent paper” without elaboration, add no value at all).

This subject is amply discussed in blogosphere. A blog called Journalology,^(2)^ and its owner, Matt Hodkginson, favor paying reviewers. Some scientists dislike the idea because funds for science are shrinking. Others think payment should be optional, e.g., if you want a faster review, you pay for it. All commenters on the blog assumed the payment would be a charge to authors, not to the journal.

One respondent suggested that if somebody takes 12 weeks to review your paper, next time that reviewer sends a paper and you are requested to review it, accept and take the same 12 weeks to do so… Another point raised by most respondents was that journals make money and thus should pay reviewers – why should reviewers work for nothing?

The same discussion has been held about participating in institutional review boards (IRBs). Participating in an IRB involves massive work without receipt of any payment. To make things even worse, such participation has liability implications. In Brazil we have a two-tier approval for research projects in medicine, the internal IRB where the research is done and the national IRB, (CONEP, the National Research Ethics Board). Brazil’s national IRB is chosen by an unusual electoral system, and it responds to the National Council of Health. Members of this council are also chosen by not widely known criteria and their meetings include payment for participation. The IRB members are not paid, but if litigation occurs – and in our litigious society we bet it will occur sooner or later – the liability probably will ensnare the IRB members.^(3)^

Hospital IRBs are closer and civil suits against its members easier. Some IRBs pay members. The University of California, San Diego, pays US$10.000 stipend for the president of its IRB and every active IRB member gets a laptop and US$500 for internet connections each year. If members resign they must return the laptop. ^(4)^

Again, jobs for free are not always jobs well done. IRBs member evaluate complex protocols, and they do it in their free time, not at their institutions. Some members do the job very well, others not so well, and others not at all. Delays, long response time and low-quality opinions are common.

In these cases, we believe, scientific papers and IRB reviewers should be educated to do such work. This is definitely not something teachers address at schools. It requires training, and yes, it should be paid. And, well paid. Working for just the honor of the work or for the prestige, is not fair – as it happens – just for sitting in the meetings but not doing much more…
