# *Erratum:* Paying reviewers for scientific papers and ethical committees

**DOI:** 10.1590/S1679-45082014ED3259E

**Published:** 2017

Jacyr Pasternak^1^,

Sidney Glina^1^

^1^Hospital Israelita Albert Einstein, São Paulo, SP, Brazil.

In the editorial “Paying reviewers for scientific papers and ethical committees” DOI number 10.1590/S1679-45082014ED3259, published at einstein (São Paulo). 2014;12(3):vii-ix, pages viii and ix, reference 2 was wrongly cited. We cited “Should peer reviewers be paid for their work?” [Internet]. [moderated by David Poeppel and Greg Hickcok]. post on 2011 Jan 29. [cited 2014 Sep 12]. Available from: http://www.talkingbrains.org/2011/01/should-peer-reviewers-be-paid-for-their.html. However the correct citation should be “Why reviewers decline, and paying for peer review” http://journalology.blogspot.co.uk/2007/01/why-reviewers-decline-and-paying-for.html.

Also we stated that Matt Hodgkinson, the owner of the blog, favored paying reviewers. However it is written on the blog: ”I’m not sure that I agree that payment would fail to act as an incentive, but I do have doubts that journals should move to making payments.”, which it is not an endorsement of paying to review.

