# Supplementary material for: Paying reviewers for scientific papers and ethical committees
Source: Einstein (Sao Paulo). 2014 Jul-Sep;12(3):7–15. doi: 10.1590/S1679-45082014ED3259 (PMC4872933; doi:10.1590/S1679-45082014ED3259)
Supplement: Translation

# Translation: Pagamento para revisores de artigos científicos e comitês de ética

**Authors:** Jacyr Pasternak, Sidney Glina

**Affiliations:** 1 Hospital Israelita Albert Einstein, São Paulo, SP, Brasil.

Richard Smith, ex-editor do *British Medical* *Journal*, diz que a revisão por pares é difícil de definir em termos operacionais,^(1)^ porém quase todos concordam que esse tipo de revisão é primordial para a prática da ciência. Esse processo pode ser conduzido de diversas maneiras. O editor, por exemplo, pode enviar o artigo para dois amigos que conhecem, ou não, o assunto. Se ambos forem favoráveis à publicação do artigo, este é publicado; porém se ambos forem contra, o editor é quem decide ou envia a um terceiro amigo para definir a questão (os editores, no início de suas carreiras, possuem muitos amigos, porém acabam perdendo muito deles após algum tempo no “ramo”). No artigo de Smith sobre revisão por pares publicado no *Journal of the Royal Society of Medicine*, há a menção a Robbie Fox, ex-editor da *Lancet*, que não era um admirador desse sistema de revisão e brincava dizendo que a *Lancet *escolhia os artigos que seriam publicados atirando todos manuscritos escada abaixo, sendo publicados aqueles que chegassem ao fim da escada. Além disso, o Dr. Smith cita que “uma revisão sistemática de todas as evidências sobre revisão por pares concluiu que essa prática é baseada, principalmente, na fé, ao invés de fatos...”

Todos sabem dos problemas que envolvem a revisão por pares. Ela é lenta: aqueles que concordam em realizá-la, o fazem em seu tempo livre. A maioria dos revisores não têm muito tempo livre, já que está sobrecarregada com pedidos de novas revisões, além de outras atividades. Ela é inconsistente: alguns artigos submetidos a um periódico são considerados excelentes enquanto outros nem mesmo são considerados para serem revisados por pares. Há muitos vieses e eles não são evitados pela revisão anônima. Na verdade, se os revisores são bem escolhidos e estão na área adequada, podem facilmente identificar os autores do artigo em questão ou, ao menos, a instituição a que eles pertencem. Alguns autores têm um estilo pessoal e, ao ler seus artigos, é possível identificá-los. Aos autores de instituições com menos prestígio – ou, por exemplo, que pertencem a América Latina ao invés do mundo civilizado – tem se o efeito Mateus: “àqueles que têm tudo, tudo deve ser dado; àqueles que não têm nada, o pouco que têm deve ser tirado deles”. Em outras palavras, aqueles que pertencem a essas instituições ou que não são conhecidos pelos revisores têm mais dificuldade de publicarem. Resultados negativos são difíceis de publicar, mesmo quando confrontam o conhecimento convencional, isto é, algo que todos conhecem como correto, mas que nunca foi e que, algumas vezes, não está realmente correto.

Apesar disso, estamos presos à revisão por pares. É como a democracia: um sistema terrível, porém melhor do que todas as alternativas testada em relação a ela. Muitas sugestões têm sido feitas para melhorar o sistema de revisão por pares e uma que deve ser considerada é o pagamento dos revisores. Eles devem ser profissionais e qualificados; ninguém nasce um revisor. A revisão de artigos leva tempo e requer muito trabalho. O argumento contra o pagamento de revisores define a ciência como uma comunidade de acadêmicos que revisa hoje para ser revisada amanhã, sempre sem custo. Tal como uma “assistência médica gratuita”, que parece ser gratuita, mas não é, já que no final alguém paga a conta.

Muitos periódicos cobram os pesquisadores quando seus artigos são revisados, mas poucos pagam seus revisores. Isso acontece, principalmente, mas não somente, com artigos submetidos em periódicos *open access*. Se considerarmos revisores como profissionais, eles devem ser qualificados para o trabalho, e sim, deveriam ser pagos. O total pago pode ser simbólico, e os revisores não sobreviveriam a partir disso, porém seria um valor considerável. E, portanto, se pagos, poderiam realizar um trabalho mais cuidadoso e cumprir os prazos – revisões que consistem em comentários como “artigo excelente” sem sugestões de melhoria, definitivamente, não agregam valor.

Esse assunto é amplamente discutido em *blogs*. Um *blog* chamado *Journalology,* ^(2)^ e seu administrador, Matt Hodkginson, são a favor do pagamento de revisores. Alguns pesquisadores desaprovam a ideia devido ao fomento da ciência estar diminuindo. Outros defendem que o pagamento deve ser opcional, por exemplo: se quer uma revisão rápida, você deve pagar por isso. Todos participantes do *blog* concordam que o pagamento deve ser feito pelos autores, e não pelo periódico.

Um participante sugeriu que se um alguém leva 12 semanas para revisar seu artigo, no próximo, quando esse revisor enviar um artigo para sua revisão, aceite e revise nas mesmas 12 semanas. Outro ponto enfatizado pela maioria dos participantes foi que os periódicos têm receita e, portanto, devem pagar os revisores – por que os revisores devem trabalhar sem compensação?

A mesma discussão tem sido feita em relação a participação em conselhos de revisão institucional (CRIs). A participação em CRI envolve muito trabalho sem nenhuma compensação financeira. Para piorar as coisas ainda mais, tal participação implica “responsabilidade”. No Brasil, temos um sistema bipartido para aprovação de projetos de pesquisa em medicina, o CRI interno, no qual a pesquisa é realizada, e o CRI nacional (o CONEP, Comissão Nacional de Ética em Pesquisa). O CRI nacional é escolhido por meio de um sistema eleitoral incomum e reporta-se ao Conselho Nacional de Saúde. Os membros do conselho são também escolhidos por um critério não muito conhecido, e as reuniões do grupo incluem pagamento para participação. Os membros do CRI não são pagos, porém quando ocorre litígio – e em nossa sociedade litigiosa, isso acontece cedo ou tarde – o litígio “pega” os membros do CRI.^(3)^

O CRI de um hospital é mais próximo e processar seus membros é mais fácil. Alguns CRIs pagam seus membros. A Universidade da Califórnia, em San Diego, paga US$10 mil ao presidente de seu CRI, além de cada membro ativo receber um laptop e US$500 anualmente para cobrir custos com acesso a internet. Se um membro renúncia, o laptop deve ser devolvido.^(4)^

Novamente, trabalhos gratuitos nem sempre são bem feitos. Os membros do CRI avaliam protocolos complexos, fazem essa atividade em seu tempo livre e fora de suas instituições. Alguns membros fazem o trabalho muito bem, outros não tão bem e outros não fazem bem. Atrasos, demora nas respostas e avaliações de baixa qualidade são comuns.

Acreditamos que nos casos, revisores de artigos científicos e membros de CRI devem ser qualificados para realizar essa atividade. E que tal qualificação, definitivamente, não é algo abordado na universidade. Para tornar-se um revisor é necessário treinamento, e, sim, a revisão deve ser paga e bem paga. O trabalho somente pela honra e prestígio, não é justo – como acontece hoje – assim como sentar-se em reuniões e não fazer muito mais do que isso...
